# Unusual Case of Ischemic Colitis Caused by Low-Dose Sumatriptan Therapy in a Generally Healthy Patient After Strenuous Physical Activity

**DOI:** 10.7759/cureus.17125

**Published:** 2021-08-12

**Authors:** Swann Tin, William Lim, Rushaniya Umyarova, Marutha Arulthasan, Magda Daoud

**Affiliations:** 1 Internal Medicine, Richmond University Medical Center, Staten Island, USA; 2 Internal Medicine/Gastroenterology, Richmond University Medical Center, Staten Island, USA

**Keywords:** ischemic colitis, sumatriptans, large bowel necrosis, serotonin agonists, migraine disorder

## Abstract

Ischemic colitis refers to an inflammatory condition of the large bowel caused by ischemia. It usually presents with an acute onset abdominal pain followed by hematochezia. It can occur as a result of arterial occlusion (embolic or thrombotic), venous thrombosis, or hypoperfusion of mesenteric circulation secondary to dehydration, surgery, or medications. Herein, we present an unusual case of sumatriptan-induced ischemic colitis. Sumatriptan succinate is a selective serotonin (5-hydroxytryptamine-1) receptor agonist that is usually prescribed for refractory migraine headaches. This is a 59-year-old female who presented with acute onset abdominal pain followed by bloody diarrhea after vigorous physical activities. She has a past medical history of non-specific colitis (one time, 15 years ago) and chronic migraine for which she was on low-dose sumatriptan therapy (one tab once or twice a week). On the day of the event, the patient took sumatriptan in the morning and had strenuous activities throughout the day, and overnight she developed abdominal pain. It was followed by bouts of bloody diarrhea. The colonoscopy revealed erythematous mucosa with significant ulceration and necrosis involving the distal transverse colon, splenic flexure, descending colon, and proximal colon, suggestive of ischemic colitis. Unlike previously reported cases, this patient was only on low-dose sumatriptan therapy without frequent dosing. So, her risk of ischemic colitis from triptan therapy could have been accelerated by excessive sweating and strenuous physical activities. The patient was treated with intravenous hydration, bowel rest, intravenous antibiotics, and withdrawal of sumatriptan and her condition improved within the next two to three days.

## Introduction

Ischemic colitis (refers to an inflammatory condition of the large bowel secondary to ischemia. It can be related to acute arterial occlusion, venous thrombosis, or hypoperfusion of the mesenteric circulation from severe dehydration, surgery, or medications. Sumatriptan succinate is a selective serotonin (5-hydroxytryptamine-1) receptor agonist, which is widely used for migraine headaches. Sumatriptan relieves migraine by vasoconstriction of intracranial blood vessels but at the same time, it can cause vasospasm of coronary blood vessels, myocardial infarction, and less frequently bowel ischemia.

## Case presentation

A 59-year-old female with a past medical history of migraine headache and nonspecific colitis 15 years ago presented to the emergency room (ER) for severe abdominal pain and bloody diarrhea. The pain was intermittent, colicky in nature, 8/10 in intensity which later became more diffuse and persistent. Prior to this, she was in good health and did exercise three times a week. She denied any history of hypertension, hypercholesterolemia, diabetes mellitus, or heart disease. She was on acetaminophen and low-dose sumatriptan for migraines. Her headache was associated with weather changes or stress and she normally took sumatriptan once a week and rarely twice a week. On the same day, the patient took one tablet of sumatriptan and she went biking with her husband in the afternoon. They spent a few hours of biking and she was sweating excessively throughout the whole time. She was exhausted and started to feel nauseated at bedtime. Later, she threw up and developed abdominal pain. Diarrhea began in the morning, which was watery but later she noticed some fresh blood and blood clots in it.

Upon arrival to ER, she was afebrile, and her vital signs were normal. She seemed a bit uncomfortable because of the pain. She was given intravenous (IV) hydration and IV morphine for the pain. Her initial hemoglobin was 15 g/dL, which went down to 14 g/dL with IV hydration. Her lungs are clear. Heart sounds are regular with no murmur or additional sounds. The abdomen was soft but diffusely tender to palpation without rebound tenderness. Initial laboratory studies were normal except for an elevated white cell count of 14.4 k/µL.

During her ER stay, the patient was prescribed bowel rest and IV metoclopramide for nausea and vomiting. Computed tomography (CT) abdomen revealed an inflammatory change in distal transverse, descending, and sigmoid colon consistent with nonspecific colitis vs IC. IV levofloxacin and metronidazole were initiated, and she was planned to have esophagogastroduodenoscopy (EGD) and colonoscopy on the next day.

**Figure 1:**
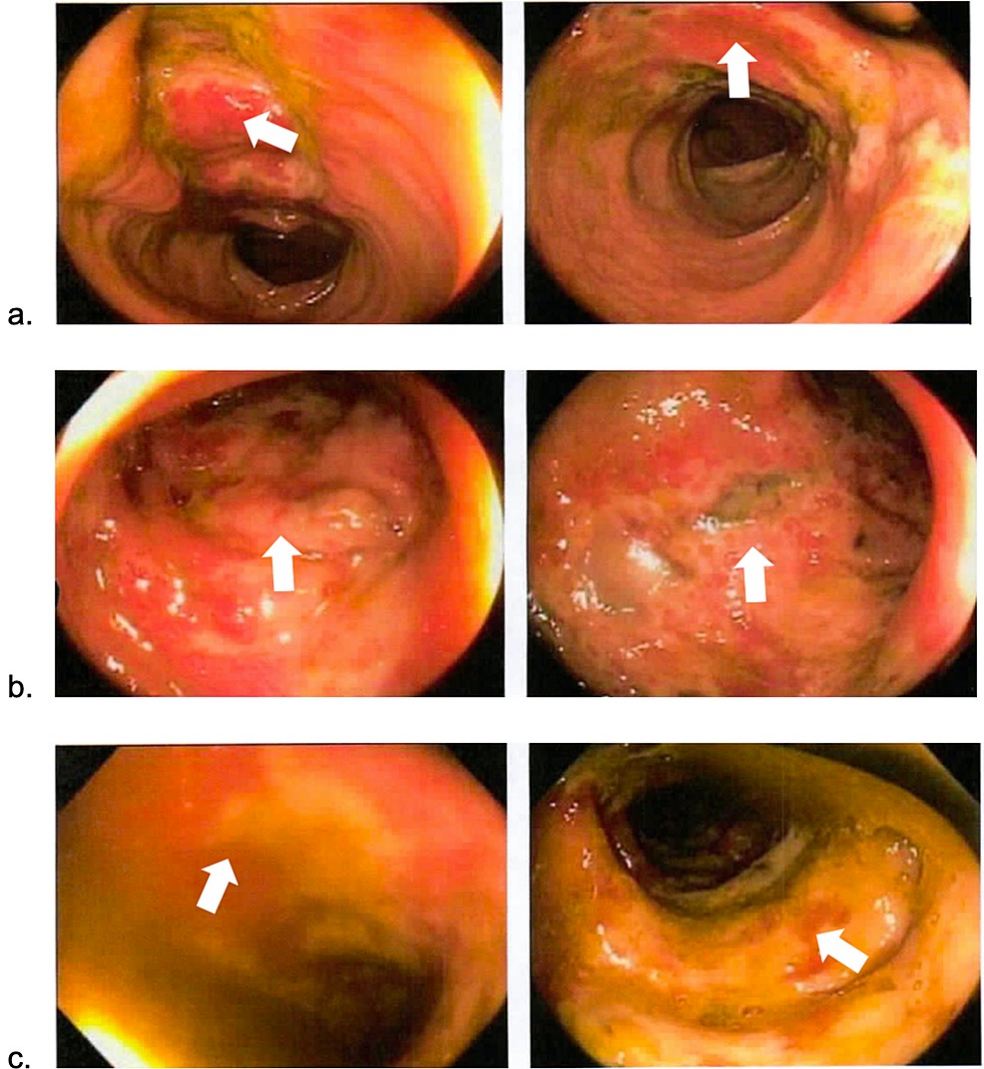
Colonoscopy revealing erythematous mucosa with significant ulceration and necrosis (white arrows). (a) Ascending colon, (b) transverse colon, and (c) descending colon.

**Figure 2:**
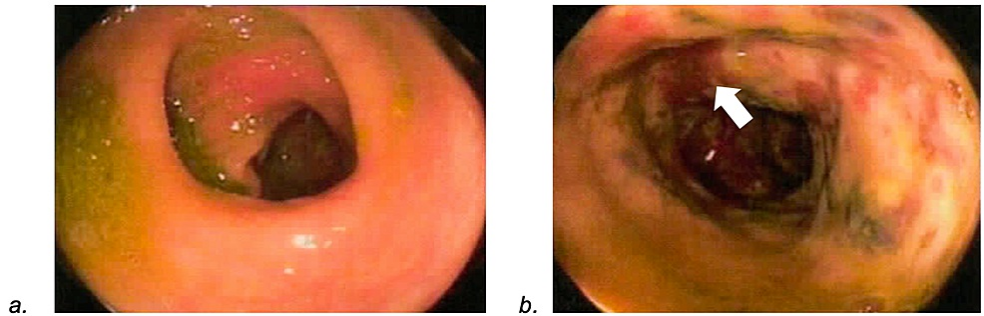
Colonoscopy revealing (a) normal sigmoid colon versus (b) area of colitis showing erythematous mucosa with significant ulceration and necrosis (White arrows).

The EGD report showed diffuse erythematous mucosa in the antrum and body of the stomach, suggestive of gastritis. Her colonoscopy revealed erythematous mucosa with significant ulceration and necrosis involving some area of ascending colon (Figure 1a), distal transverse and splenic flexure (Figure 1b), descending colon (Figure 1c), and up until sigmoid colon (Figure 2), suggestive of ischemic colitis.

On the next day, her abdominal pain improved, and a clear liquid diet was initiated. An echocardiogram was ordered to exclude any vegetations and embolic cause of IC but it was normal. Her diet was slowly advanced to a regular diet and her nausea and abdominal pain were resolved by the time of discharge.

Before her discharge, the patient was educated that her bowel ischemia and inflammation were most likely caused by reduced blood supply due to transient narrowing of colonic blood vessels as a result of sumatriptan use. The ischemia was again precipitated by hypotension and hypoperfusion from severe dehydration and strenuous physical activity. She was also instructed to discontinue sumatriptan and to follow up with her neurologist for an alternative medication.

## Discussion

IC occurs when blood flow to areas of the large intestine is temporarily reduced causing bowel ischemia, inflammation, and necrosis [1]. Common causes include embolism (50%), thrombosis, and nonocclusive ischemia. Nonocclusive ischemia is due to vasoconstriction of splanchnic vessels leading to hypoperfusion; usually involving the watershed areas of the colon (splenic flexure and rectosigmoid junction) because of limited collateral circulation [2]. Nonocclusive ischemia usually results from severe hypotension, dehydration, surgery, trauma, or medications. Drug-induced IC is less common compared to other causes and is usually related to antihypertensives, nonsteroidal anti-inflammatory drugs (NSAIDs), oral contraceptive (OC) pills, digoxin, vasoconstrictors (e.g., ergotamine products), and cocaine [3,4].

Sumatriptan succinate is a selective serotonin (5-hydroxytryptamine-1) receptor agonist that works by narrowing the intracranial blood vessels and interfering with the release of chemical neurotransmitters that causes the symptoms of migraine headache [5]. It also has an action on both systemic and coronary circulations and can cause serious complications such as coronary vasospasm, myocardial infarction, and bowel ischemia [6,7]. There are few case reports suggesting the association between serotonin receptor agonists and IC but most of them have a confounding bias as patients have other comorbid conditions or are taking NSAIDs, OC pills.

This case is distinctive from others for many reasons. The patient does not have any significant past medical history except for migraines and one-time nonspecific colitis. In her case, she does not take NSAIDs since they do not alleviate her pain, only sumatriptan brings mild to moderate relief. The patient also lacks risk factors for vascular disease, including hypertension, diabetes mellitus, hypercholesterolemia, tobacco, or OC pills use. Most importantly, she does not have frequent sumatriptan use (only once a week) and lacks multiple dosing of sumatriptan seen in previous cases.

In general, IC presents with nausea, vomiting, abdominal pain, diarrhea, and hematochezia, however, early signs and symptoms are nonspecific. The initial test of choice is CT angiography since it is highly accurate in diagnosing mesenteric ischemia and is also helpful to exclude other possible causes of acute abdominal pain [8]. The diagnosis is then confirmed with colonoscopy or sigmoidoscopy, which allows the direct visualization of bowel revealing edema, erythema, submucosal hemorrhage, and epithelial necrosis [9]. Colonoscopy is also useful for excluding other differential diagnoses such as diverticular diseases, inflammatory bowel diseases, or neoplasm.

IC caused by medications is a transient condition and the symptoms usually recover in two to three days with the removal of the offending agent. Initial management includes bowel rest, fluid resuscitation, hemodynamic monitoring, pain control, and initiation of broad-spectrum antibiotics [10]. However, in rare cases, colitis can progress to extensive bowel necrosis, persistent bleeding, peritonitis, and the development of intestinal strictures [11]. Even though the majority of cases have a complete recovery after eliminating the inciting medications, some patients may require surgical removal of the affected bowel segment to minimize adverse outcomes [12].

## Conclusions

5-HT 1 receptor agonists (Sumatriptan) are generally prescribed by primary physicians for migraine headaches that are not responding to initial NSAID therapy. Although this patient had a complete recovery, IC can result in significant morbidity and adverse outcome. It is important to review the risks and benefits before prescribing any medication and to educate the patients regarding possible complications. This case report is intended to demonstrate an unusual adverse event and to promote an increased awareness of IC, which can occur with low doses of triptan drugs.
